# A novel AP/PA total body irradiation technique using abutting IMRT fields at extended SSD

**DOI:** 10.1002/acm2.14213

**Published:** 2024-02-29

**Authors:** Xiaomin Liang, Peixiong Li, Qiuwen Wu

**Affiliations:** ^1^ Medical Physics Graduate Program Duke Kunshan University Kunshan Jiangsu China; ^2^ Department of Radiation Oncology Duke University Medical Center Durham North Carolina USA

**Keywords:** extended SSD, field matching, intensity modulated radiation therapy, total body irradiation

## Abstract

**Purpose:**

To develop a Total Body Irradiation (TBI) technique using IMRT at extended SSD that can be performed in any size Linac room.

**Methods:**

Patients studied were placed on a platform close to the floor, directly under the gantry with cranial‐caudal axis parallel to the gantry rotation plane and at SSD ∼200 cm. Two abutting fields with the same external isocenter at gantry angles of ±21˚, collimator angle of 90˚, and field size of 25 × 40 cm^2^ are employed for both supine and prone positions. An iterative optimization algorithm was developed to generate a uniform dose at the patient mid‐plane with adequate shielding to critical organs such as lungs and kidneys. The technique was validated in both phantom and patient CT images for treatment planning, and dose measurement and QA were performed in phantom.

**Results:**

A uniform dose distribution in the mid‐plane within ±5% of the prescription dose was reached after a few iterations. This was confirmed with ion‐chamber measurements in phantom. The mean dose to lungs and kidneys can be adjusted according to clinical requirements and can be as low as ∼25% of the prescription dose. For a typical prescription dose of 200 cGy/fraction, the total MU was ∼2400/1200 for the superior/inferior field. The overall treatment time for both supine/prone positions was ∼54 min to meet the maximum absorbed dose rate criteria of 15 cGy/min. IMRT QA with portal dosimetry shows excellent agreement.

**Conclusions:**

We have developed a promising TBI technique using abutting IMRT fields at extended SSD. The patient is in a comfortable recumbent position with good reproducibility and less motion during treatment. An additional benefit of this technique is that full 3D dose distribution is available from the TPS with a DVH summary for organs of interest. The technique allows precise sparing of lungs and kidneys and can be executed in any linac room.

## 1 INTRODUCTION

Total Body Irradiation (TBI) has long been used for the treatment of hematologic malignancies to irradiate blood‐related cancer cells like leukemia, lymphomas, and myelomas.^1^, ^2^, ^3^, ^4^ TBI aims to deplete a patient's bone marrow and suppress the immune system by delivering a uniform dose to the entire body with megavoltage photon beams. To immunosuppress the patient and avoid rejection of the donor bone marrow, the patient needs to receive the TBI treatment before the bone marrow transplantation. For certain kinds of diseases, the patient receives a chemotherapy regimen before TBI treatment.^5^ In standard radiation therapy of local regional disease, each field could easily deliver a dose to the whole target; however, placing the entire target (patient whole body) for TBI in one single field is a challenge. To meet the criteria, TBI was traditionally carried out when the patient is positioned at an extended source‐to‐skin distance (SSD), usually over 400 cm, in a large linac treatment room.^6^

Different institutions have their considerations on patient positioning, treatment distance, organ‐at‐risks (OARs) protection, and other detailed setup requirement. According to AAPM Report 17, TBI methods should satisfy the requirement that the dose homogeneity inside the patient is within ±10% of the prescription dose.^7^ The two common TBI techniques use the parallel‐opposed beams, either in the anterior‐posterior (AP/PA) direction, or right‐left (RT/LT) lateral direction. In the RT/LT bilateral technique, the patient lies down on the stretcher with bending knees and is positioned to fit in a single maximum treatment field. In the AP/PA technique, the patient is positioned standing on a TBI stand and receives radiation from the AP and PA direction. In recent years, some researchers have shown that radiosensitive organs like kidneys and lungs often are damaged by systemic sclerosis (SSc) which could be caused by high radiation dose.^8^ The bilateral TBI requires more MUs than AP/PA TBI (6MV photon beams) and can result in higher maximum lung dose.^9^ Patients treated with bilateral technique without adequate kidneys and/or lung shielding may have increased radiotoxicity, but adding shielding would also compromise doses to the bones (ribs and vertebrates) in these regions. Therefore, these patients are typically treated with the AP/PA technique with partial transmission blocks for lungs and kidneys to reduce the radiotoxicity of these critical organs without significant effect on the dose to bones. However, the fabrication and quality assurance of customized partial transmission blocks and compensators are labor‐intensive and time‐consuming. Additionally, the current AP/PA TBI technique is executed in a standing position; patient anatomy could have large variations from simulation to treatment and between treatment fractions. In addition, the standing position can be challenging in maintaining acceptable setup variations for many frail patients during the long setup and treatment time.

Previous studies have demonstrated that the dosimetric quantities calculated from the commercial treatment planning system (such as Eclipse or Monaco) at the extended SSD geometry for TBI agree well with the measured TBI commissioning data.^10^, ^11^, ^12^ More studies have shown that the Eclipse Analytical Anisotropic Algorithm (AAA) algorithm is acceptable for dose calculation at the extended SSD.^12^, ^13^ As radiotherapy techniques have been developed rapidly in recent years, more institutions have utilized advanced techniques like electronic compensator (E‐comp)^14^ and IMRT,^15^, ^16^ volumetrically modulated arc treatment (VMAT),^17^, ^18^, ^19^, ^20^, ^21^, ^22^, ^23^, ^24^ and Helical TomoTherapy^25^, ^26^, ^27^, ^28^, ^29^, ^30^ in the TBI treatment. Some of these techniques use the standard SSD on Linac, and some are at extended SSD.

In this study, we developed a novel TBI technique that could be used in any C‐arm type linac room. One goal is to provide adequate sparing of lungs and kidneys by using the IMRT technique to reduce the complexity of making customized compensators and partial transmission blocks. We developed an IMRT optimization algorithm to improve the dose homogeneity and critical organ sparing. The algorithm is interfaced with Eclipse TPS for MLC leaf sequencing, dose calculation, plan evaluation, and quality assurance. A comparison between dose distribution calculated in Eclipse and measured in water phantom was performed. The treatment planning on patient CT images was also investigated.

## 2 METHODS AND MATERIALS

### 2.1 Patient setup and treatment planning

**FIGURE 1:**
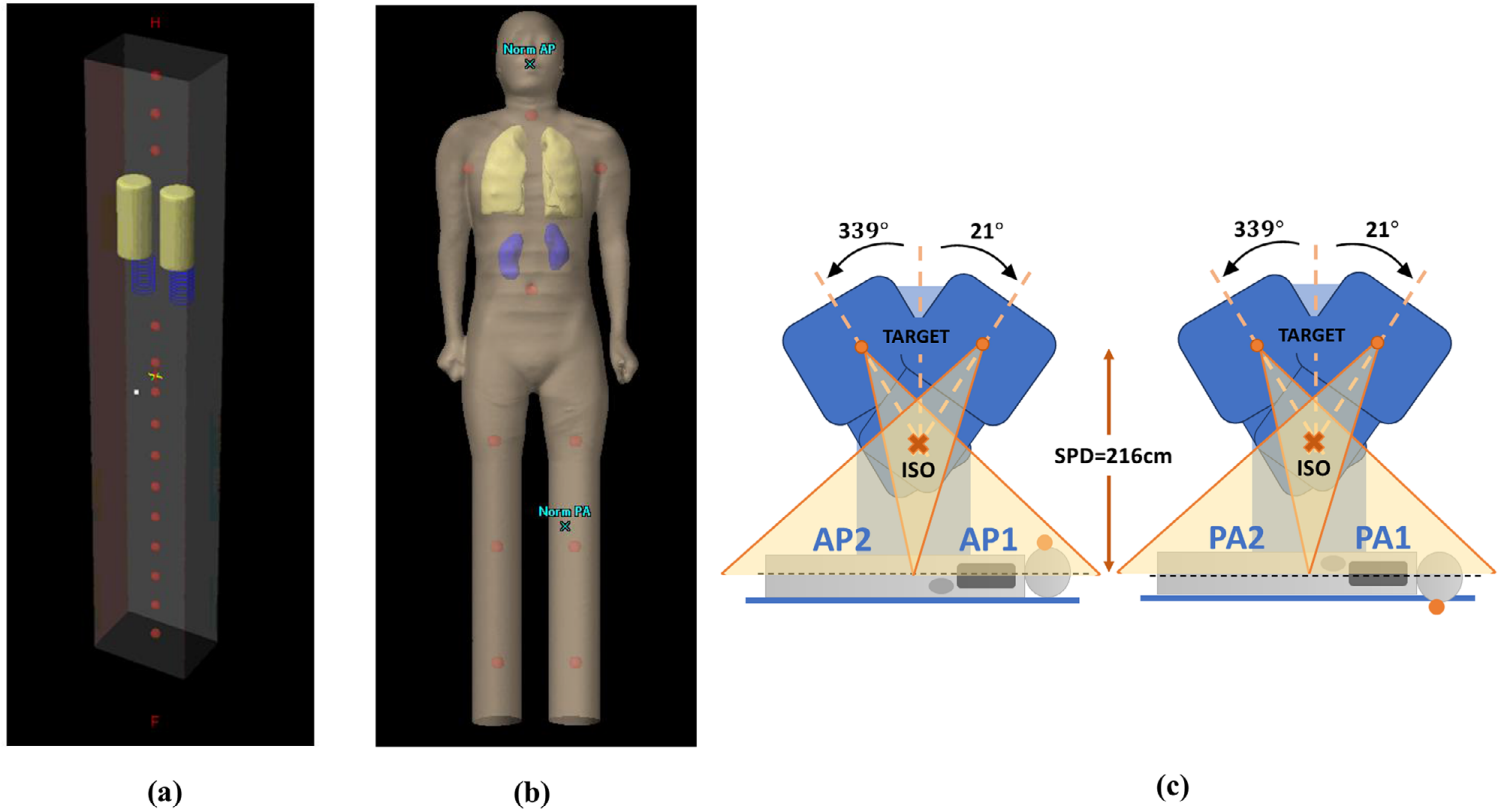
(a) The rectangular phantom with lungs (yellow), kidneys (blue), and fourteen (14) spheres (red) of 3 cm diameter used for optional dose normalization are contoured. (b) The patient CT image used in this study with leg extension, similar structures are outlined. (c) Beam arrangement and patient setup.

For the patient study, the CT image was imported into Eclipse for treatment planning, including dose calculation and plan evaluation. Usually, there is a body length limitation on the CT scanner, where the whole body cannot be scanned in one exam. Extra image processing can be performed to concatenate multiple CT scans into one image set for treatment planning. Normally patient legs and inferior portions do not have critical organs in TBI, and the anatomy is generally smooth; therefore, a common technique is to extend the anatomy below the mid‐thigh to the foot, as done in previous studies.^19^ Figure 1 shows examples of (a) phantom and (b) patient CT image with leg extension in Eclipse. For the phantom study, the simulated CT images were generated directly in Eclipse.

In both phantom and patient CT images, lungs and kidneys were contoured for planning and dose evaluation. For treatment, the patient is positioned on a platform made of polycarbonate that is placed under the gantry as close to the floor as possible with a typical SSD of ∼200 cm. The patient cranial‐caudal axis is in the gantry rotation plane. The patient is treated in both supine (AP) and prone (PA) positions with the same beam arrangements—a superior field and an inferior field, as shown in Figure 1c. Both fields in AP (or PA) share the same isocenter that is external to the patient's body and have 90° collimator rotation (so that MLC leaves move in the patient lateral direction) and field size of 25 × 40 cm^2^ (The 40 cm is in the patient Sup‐Inf direction). Therefore, the patient position does not change between these 2 subfields, which can improve the treatment efficiency and minimize positioning error. The gantry angle for the field to irradiate the superior part of the phantom or patient (Sup) is 339° and for the field to irradiate the inferior part (Inf) is 21°. The source‐to‐patient mid‐plane distance (SPD) is around 216 cm. Table 1 shows the setup information for a typical recumbent position AP/PA IMRT TBI treatment plan. For the supine patient position, the AP fields in planning and treatment are the same. For the prone position, the PA fields are symmetric between planning and delivery along the patient midplane, that is, the patient is still in the supine position, for the superior part of body the gantry angle is placed at 201° in treatment planning but 339° for the treatment.

**TABLE 1:** The setup information for a typical recumbent position AP/PA IMRT TBI treatment plan.

Beam entry direction/Patient position	AP/Supine		PA/Prone	
Field	Sup.1 (AP1)	Inf.1 (AP2)	Sup.2 (PA1)	Inf.2 (PA2)
Gantry angle (treatment)	339 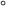	$21^{\circ }$	339 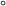	$21^{\circ }$
Gantry angle (planning)	339 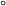	$21^{\circ }$	201 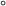	$159^{\circ }$
Field size (X × Y)	$25\times 40\;cm^{2}$ ($32\times 40\;cm^{2}$ for large patient)			
Treatment coverage	$54\times 190\;cm^{2}$ ($70\times 190\;cm^{2}$ for large patient)			
Source‐patient midline distance (SPD)	216cm			
Prescription/Fraction/Direction	100 cGy			
Typical MUs	2400	1200	2400	1200
Absorbed dose rate	<15 cGy/min			

The initial treatment plan was created in Eclipse TPS. For the fluence map (or intensity matrix as called by others), the transmission factor of 1.0 was assigned for all pixels, except for those that pass through the lungs and kidneys, where a lower value (such as 0.10) was assigned. The assignment can be easily achieved through the use of the fluence painting tool in Eclipse in the beams eye view (BEV). It is worth pointing out that the shielded lungs or kidneys are often different from the actual anatomic structure of lungs or kidneys according to the clinical protocol.^8^ For example, the shielded lung is typically smaller than the actual lungs, so relevant surrounding bony structures (clavicles, ribs, sternum, etc.) can be irradiated, and the shielded kidneys are typically expanded from the actual kidneys to provide additional protection but no medial margin to allow irradiation to the vertebrae. This is to be consistent with the current standard 3D technique.^8^ Nevertheless, the initial low value of transmission is only applied to those shielded regions easily identifiable in the BEV window. The sample prescription dose in this study was 200 cGy per fraction for 4 fractions, with a total dose of 800 cGy to the body (patient mid‐plane, usually at the umbilicus point). The shielded lungs and kidneys receive 25% of the prescription dose (200cGy total). For patients treated under SCOT protocol,^8^ the absorbed dose rate is recommended to be less than 15 cGy/min to reduce the radiotoxicity and late effect on the OARs.^31^, ^32^

The whole body dose distribution is calculated based on this initial fluence. The AAA is used for dose calculation for all cases in this study. The treatment plan including fluence maps and 3D dose distribution is exported in DICOM format as input to the in‐house built optimization program.

### 2.2 Fluence optimization

An optimization program was developed in Matlab (MathWorks, Inc., Natick, MA). The goal of optimization is to create a uniform dose on the mid‐plane of the phantom/patient and provide desired sparing to the lungs and kidneys. Given that there are only two beam incidence directions (AP and PA), the system is designed differently from the standard IMRT system. This optimization algorithm has no score or objective function and it works on the dose matrix in the mid‐plane. A ray tracing was first performed to establish the correspondence from each pixel in the fluence to the dose point on the mid‐plane; then, the dose‐to‐fluence scaling is established.

### 2.3 Optimization algorithm

**FIGURE 2:**
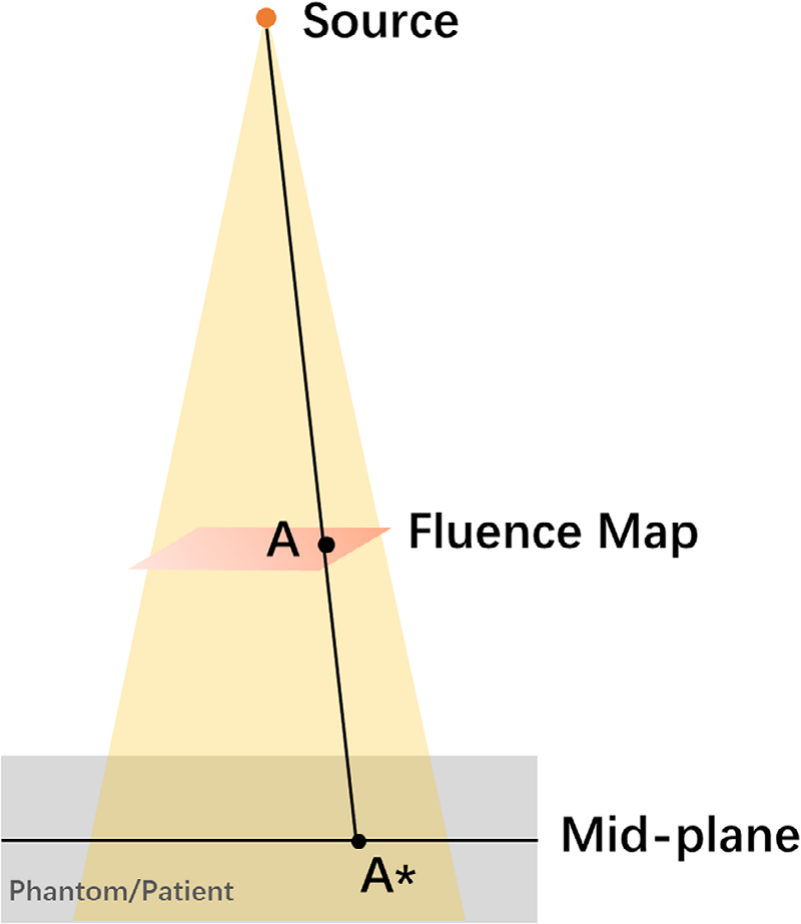
Illustration of the relative position of point A and its projection A* at the mid‐plane.

The ray tracing is shown in Figure 2, where point A in the fluence map has a fluence value of *f*_0_, and the projection of point A onto the mid‐plane of the dose matrix is A*. If the dose at point A* is *D*_A_ and the prescription dose at A* is *R_x_*, then a correction factor *CF* can be defined at this point as:

(1) $$CF\;=\frac{R_{x}}{D_{A}}\;$$

With the correction factor, the transmission factor *f_A_* at point A for the updated fluence map can be obtained as:

(2) $$f_{A}=f_{0}\times CF=f_{0}\times \frac{R_{x}}{D_{A}}$$

The updated fluence map is imported into Eclipse and then converted to a multi‐leaf collimator (MLC) leaf sequence for sliding window IMRT delivery, and subsequent dose calculation is performed.

### 2.4 MLC leaf sequence

Usually, more than one iteration is needed to achieve the goal to keep the dose difference between *D_A_* and *R_x_* less than 5%. The actual deliverable fluence after MLC leaf motion calculation could be different from the imported fluence. The typical size for the fluence map of a 25 × 40 cm^2^ field size is 108 × 168, at the grid resolution of 0.25 × 0.25 cm^2^ at the isocenter plane. The actual fluence was generated by the 60 pairs of leaves in the millennium MLCs. The MLC leaf has a resolution of 0.5 cm for the inner region and 1.0 cm for the outer region. Therefore, 2 rows of fluence pixels need to be combined to drive the 0.5 cm leaf and 4 rows of fluence pixels for the 1 cm leaf. Even though the neighboring 4 rows of transmission factors in the imported fluence map may be different, they are averaged into a single value to drive a pair of leaves, thus sharing the same value in the actual fluence. To reduce such differences, a median filter was applied for fluence map smoothing in the optimization algorithm.

The IMRT technique has the potential to reduce the hot and/or cold spots near the junction region of the two adjacent fields where the scatter from neighboring regions can be significant. This will be shown later in more detail.

### 2.5 Effective mid‐plane

Considering patient anatomy, the thickness of the patient from lateral to the medial and from head to feet can vary significantly. Moreover, the immobilization device, such as a headrest, could introduce somebody's displacement as well. This may produce an asymmetric effect for the AP and PA beams and complicate the optimization algorithms and treatment if a single absolute midplane is defined for the patient in the same way as in the phantom. To work around this, the concept of ‘effective mid‐plane’ in the patient is introduced. The effective mid‐plane is a virtual plane consisting of points inside the patient that split the patient's anterior‐posterior thickness in half, so the AP beam and PA beam will go through the same depth when arriving at this “plane.” This effective mid‐plane is not visualizable in Eclipse as it does not coincide with any physical coronal view.

**FIGURE 3:**
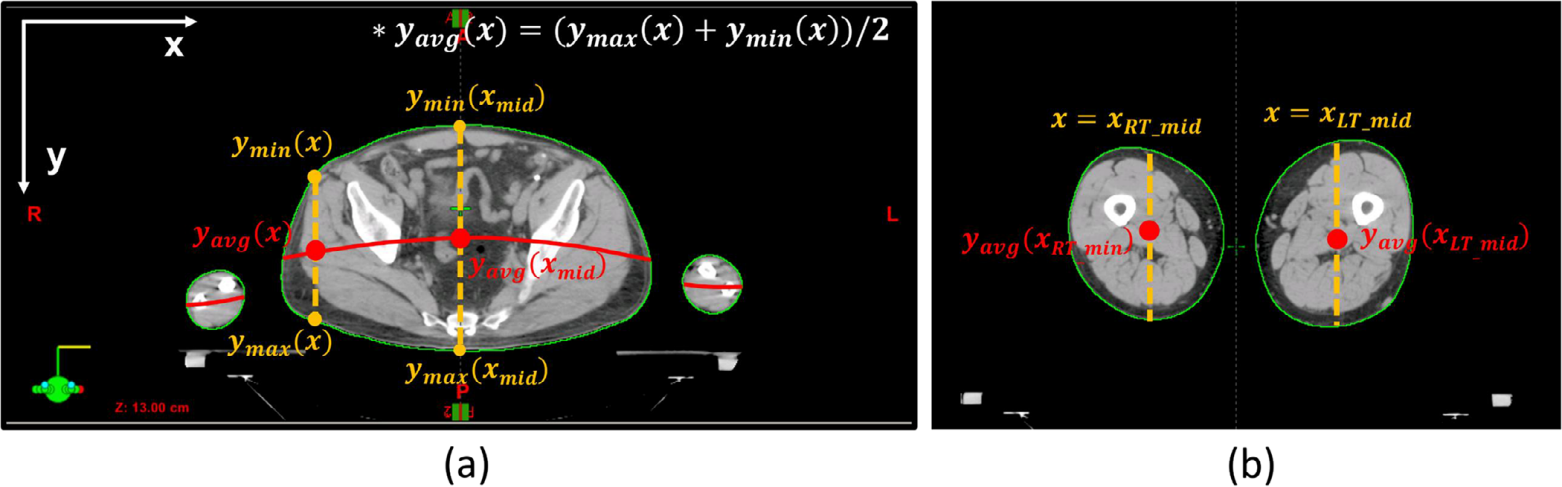
Illustration of the effective mid‐plane (red line) and the coordination of the effective dose of a CT Slice through (a) the patient's abdomen and (b) the patient's legs. The coordinate axes are labeled.

Figure 3 details the concept of the effective mid‐plane. For any line X = *x* going through the patient intersecting the patient's body contour, one could find the minimum (*y*_min_) and maximum (*y*_max_) value of the *y* coordinate of body contour at any *x* value. The average of *y*_min_(*x*) and *y*_max_(*x*) is *y*_avg_(*x*), the dose at point (*x*, y_avg_(*x*)) is assigned to the corresponding point on the effective mid‐plane that is projected by (*x*, y_avg_(*x*)) in this slice. Therefore, any *x* value at each slice has its own projected dose. Since only voxels inside the patient's body have non‐zero dose values, the dose matrices can be used for the patient body detection by assuming that the body contour was at the boundary of zero (0) and non‐zero values in the dose matrix, this is equivalent to the external body contour in Eclipse. The effective midplane is constructed using the Matlab program and no smoothing processing is performed on the midplane. For the phantom, the effective midplane is the actual midplane since it is rectangular.

### 2.6 Dose measurement and quality assurance

To verify the dose delivery accuracy, the IMRT TBI plan is delivered in a Truebeam linac to a stack of solid water phantom with embedded ion chambers. The 30 × 30 × 30 cm^3^ solid water was placed on the floor. The dose was measured by farmer chamber at 10 cm depth in the central axis of the phantom. One longitudinal mid‐line dose profile and two transverse dose profiles, going through the lungs and upper legs were measured, respectively. Since AP and PA beams were symmetric in phantom, measurement was done only for AP beams.

To investigate the feasibility of QA for TBI treatment, The IMRT QA measurement was carried out with portal dosimetry for the aforementioned treatment plan for all fields. The QA measurement was analyzed using the standard clinical parameters: 3% in dose difference, 2 mm in distance to agreement, and 10% threshold, in absolute mode.

## 3 RESULTS

### 3.1 Phantom study

**FIGURE 4:**
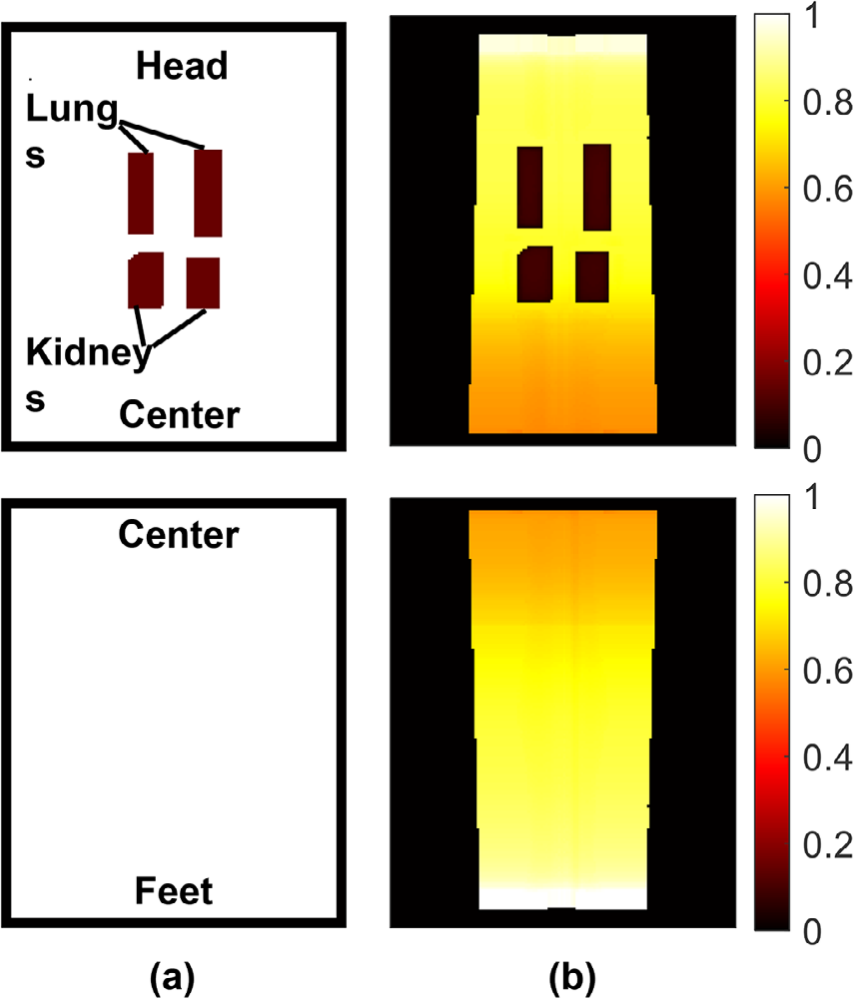
The (a) initial and (b) optimized fluence maps of AP1 and AP2 in the phantom plan. The transmission factors were in the range from 0 to 1. The fluence to the center (near the junction between the inferior field and superior field) was reduced to compensate for the oblique entry beam after optimization, largely following the inverse square law. The shielded regions of the lungs and kidneys shared a much lower transmission factor. The non‐regular fluence boundary is the result of the varying distance to the source, that is, the distance near the center region is smaller than the head and feet region.

The initial and optimized fluence maps for the phantom are shown in Figure 4. The shielding for lungs and kidneys is outlined based on the lungs and kidneys contour. Skin flash was not added in the phantom study. The gradual change in transmission factor along the Sup‐Inf direction in the optimized fluence map is to compensate for the oblique beam entry to improve dose homogeneity.

**FIGURE 5:**
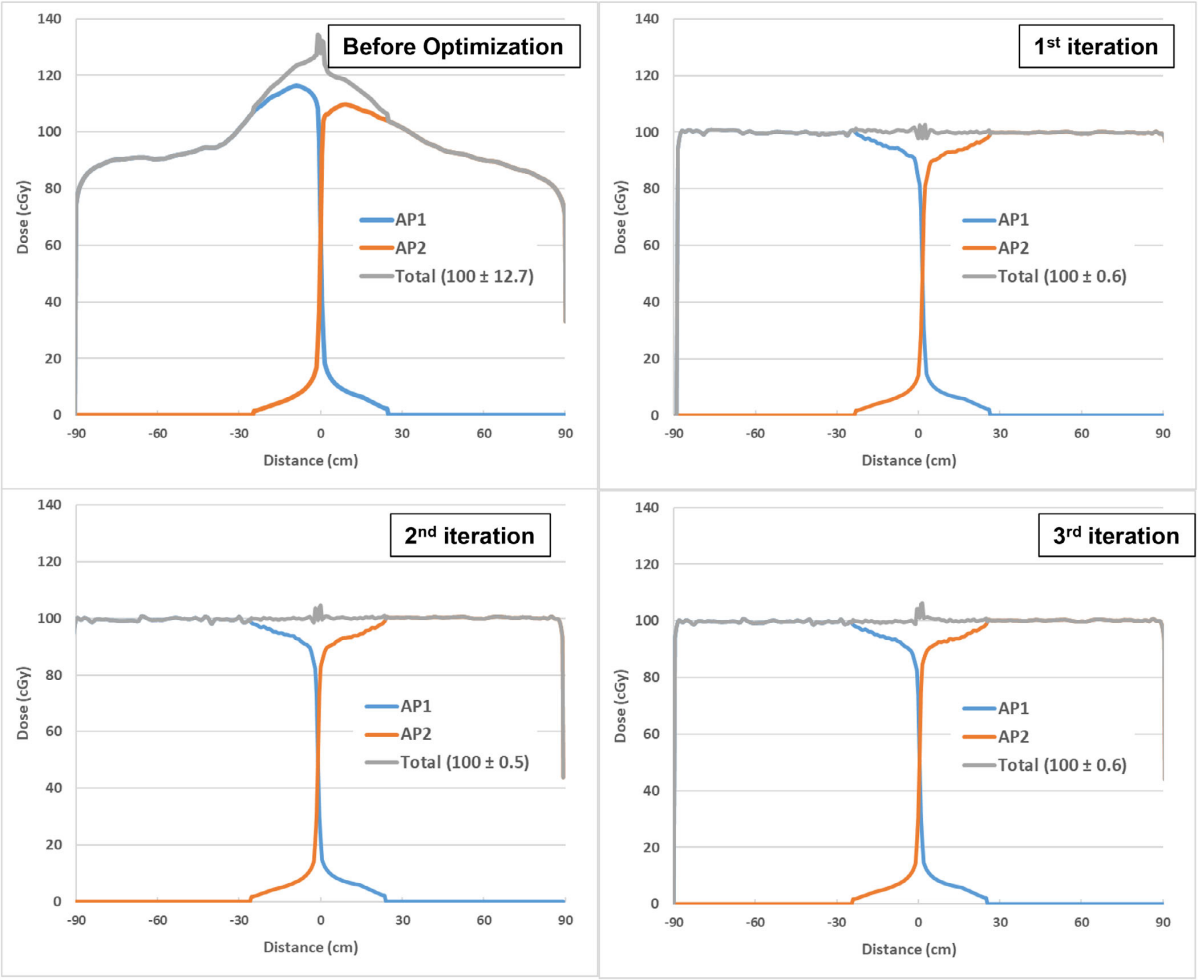
The mid‐line dose profiles for phantom before and after optimization. Blue and orange lines are the dose profiles for AP1 and AP2 fields. The grey line is the total dose profile with the contribution from AP1 and AP2. The numbers inside the parenthesis are mean ± stdev. After 1st iteration, the mid‐line dose difference for the phantom was reduced to within ±1% of the prescription dose, further iterations improved little for the phantom.

The mid‐line dose profile for phantom before optimization, after the first, second, and third iterations, are shown as grey lines in Figure 5. The profiles improved significantly after 1st iteration and stabilized at 2nd and 3rd iterations. The fluctuations near the center are caused by the field abutting effect, which is very small in size and negligible in the phantom measurement.

In Figure 5, the variations in the mid‐line dose profile before optimization was over ±12% of the prescription dose. After the first iteration, the dose difference calculated by the imported optimal fluence map becomes less than ±1% of the prescription dose. The variation after the second and third iterations did not improve much, probably due to the smooth phantom. The fluctuation in dose profiles near the central regions was caused not only by the phantom scatter from the abutting fields (blue and orange lines in Figure 5) but also by the difference in the transmission values between the optimal fluence and the actual fluence due to the MLC leaf motion calculation, as they are at the edge of the MLC. Except for the fluctuation near the center, the overall dose profile is much more uniform and closer to the prescription dose.

**FIGURE 6:**
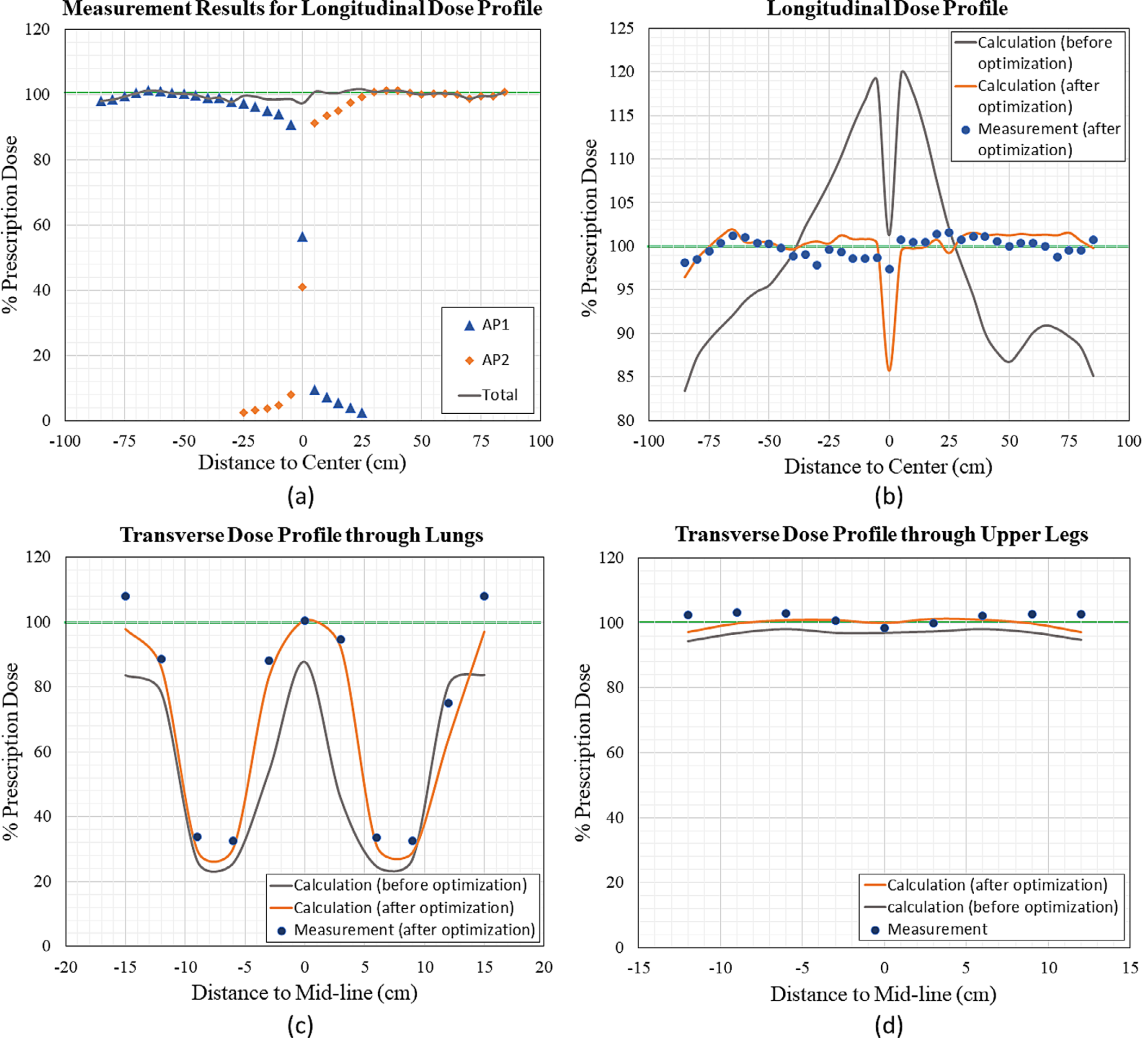
The measured dose profiles for phantom. The green lines on each graph show the 100% prescription dose. Calculation and measurement agree well in both longitudinal (patient sup‐inf) and transverse (patient med‐lat) directions.

One longitudinal mid‐line dose profile along the sup‐inf direction, and two transverse dose profiles, going through the lungs and upper legs respectively, are measured. The measurement was performed on Truebeam Linac with solid water phantom and farmer chamber. Figure 6a shows the measurement data for AP1 and AP2 fields separately. The scatter dose from the adjacent field was significant (>2% prescription dose) even at 25 cm away from the field junction. The comparison between the measurement results and the calculation results before and after optimization for the longitudinal dose profile is shown in Figure 6b. For the plan before optimization, the dose difference exceeded ±20% prescription dose (from 83% to 120% prescription dose). For the plan after the second iteration, the absolute dose difference calculated by Eclipse had reduced to within 5% of the prescription dose (from 96.5% to 102% prescription dose). The dose profiles for two transverse dose profiles, going through the lungs and upper legs, are shown in Figure 6c,d. From the dose profile through the lungs, optimization kept the lung dose at around 25% prescription dose, and increased the dose to around 100% prescription dose at the surrounding region of the lungs. We want to point out that in the phantom plan, the lung was assigned a water density, so they can be compared with measurement. The “calculation before optimization” was based on the initial arbitrarily set fluence values of 0.1 to lungs/kidneys.

### 3.2 Patient study

**FIGURE 7:**
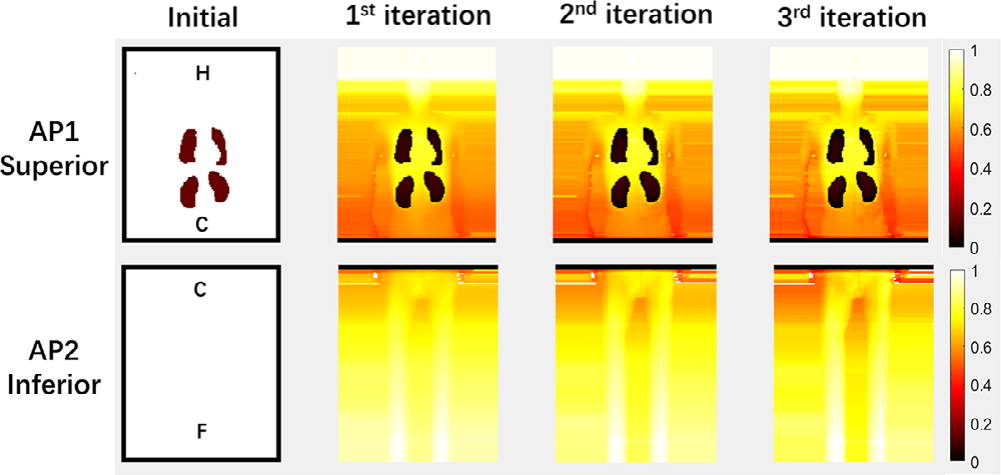
The fluence maps for each field in AP before and during optimization. The relative position of the head (H), center (C), and feet (F) of the patient were marked. The shielding to the lungs and kidneys was seen. The fluence to the center was reduced to compensate for the oblique entry beam after optimization.

**FIGURE 8:**
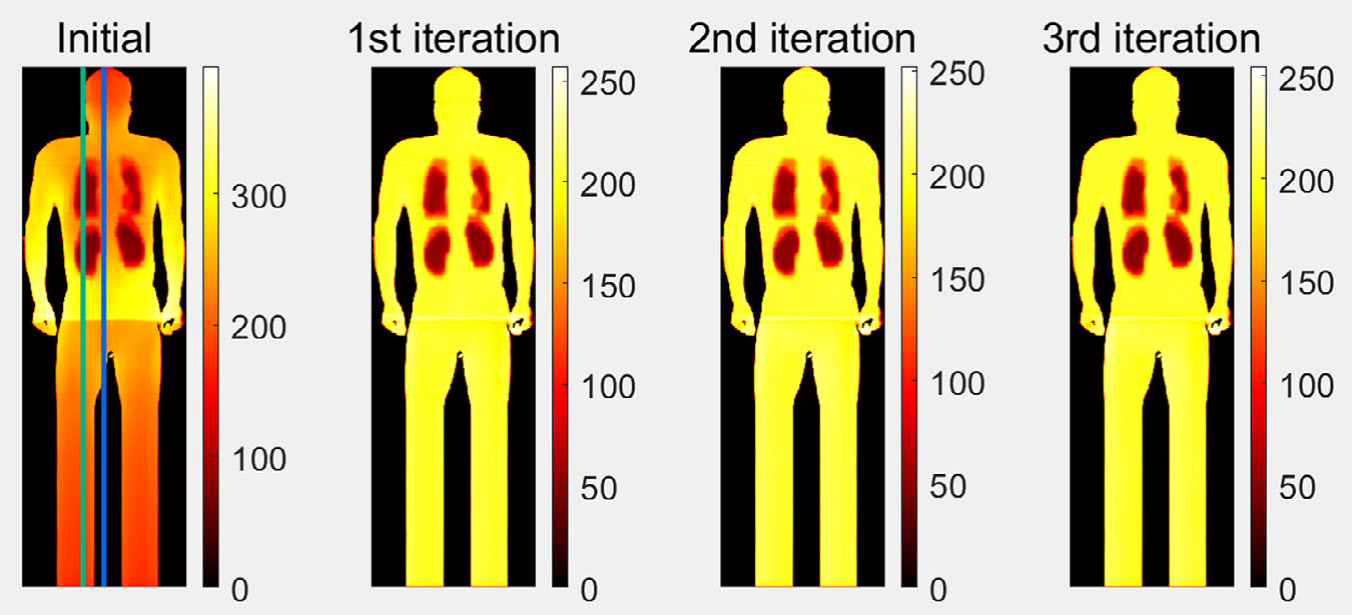
The effective mid‐plane dose matrix for patients before and during optimization. Notice different color map scales are used in the Initial and other subplots to show more details in dose distribution. Optimization increased the overall dose uniformity. Lungs and kidneys are spared as prescribed. Green and blue vertical lines indicate the position of the dose profile in Figure 9.

**FIGURE 9:**
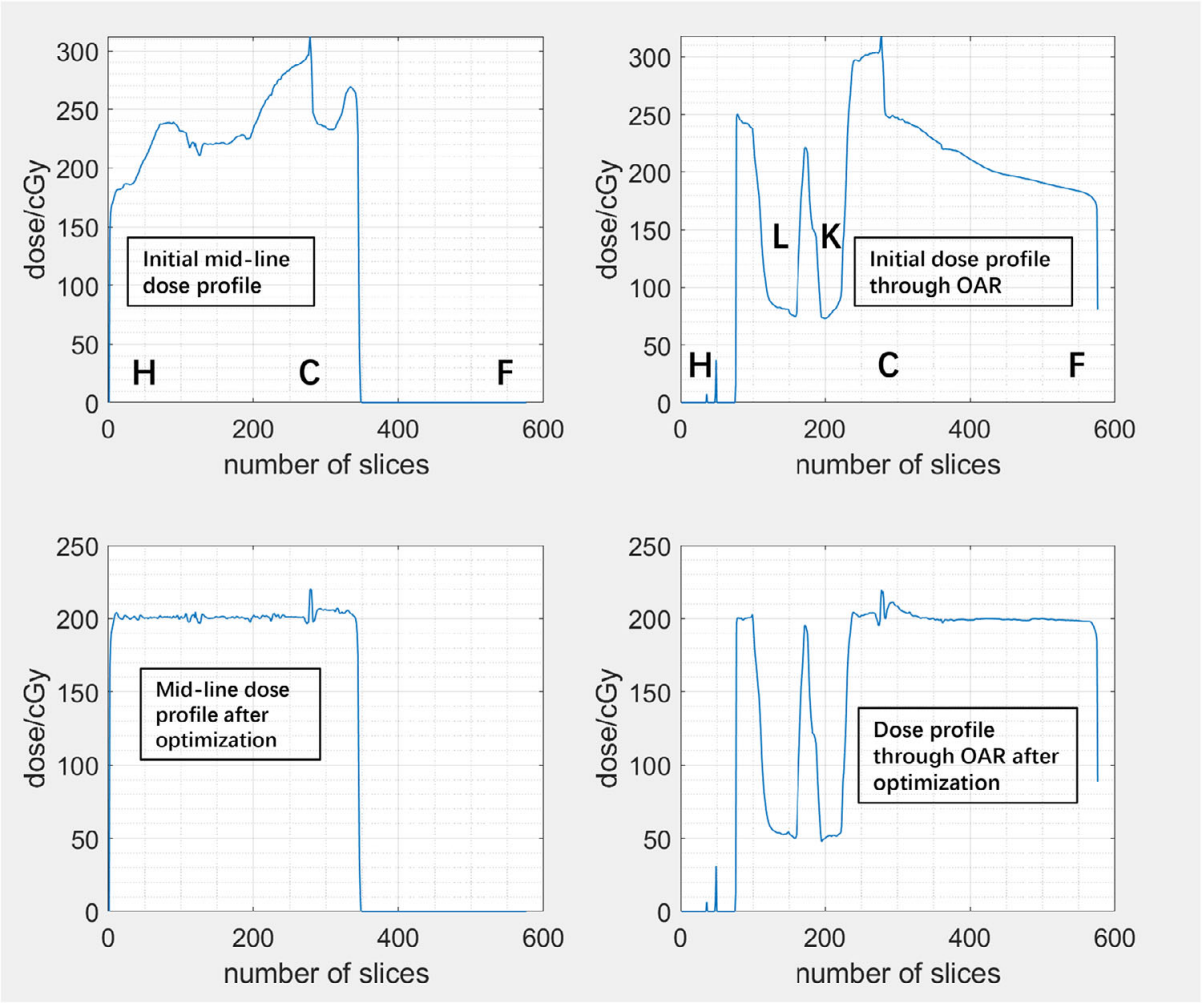
The dose profile extracted from the effective mid‐plane dose matrix through patient mid‐line (blue line in Figure 8) and OARs (green line in Figure 8). The relative position of the head (H), the center of the patient (C), feet (F), and the shielding to the lung (L) and kidney (K) were labeled. Except for the high dose near the field junction in the center region, the mid‐line dose profile and the dose profile through the legs were close to 100% prescription dose with small variations. Kidneys and lung doses were kept at around 25% to 30% of the prescription dose.

The planning process for patient is similar to the phantom, except the following extra steps that were performed for patient case only: effective midplane and skin flash. To account for the patient's movement during treatment and reduce the unnecessary irradiation, skin flash was applied to the treatment plans, by filling the fluence outside the body with the same value at the body. This is consistent with the current 3D technique that a maximum open field is used for each direction. The fluence maps for each field before and after optimization are shown in Figure 7. The relative position of the head (H), center (C), and feet (F) of the patient were marked. Optimization reduced the transmission factor gradually when getting closer to the center of the patient. However, transmission factors around the lung level kept the maximum value to increase the dose near the lung shielding. The effective mid‐plane dose matrix for the treatment plan is shown in Figure 8. The dose homogeneity had been improved after optimization. The dose profiles were shown in Figure 9 through patient mid‐line (blue line in Figure 8) and OARs (green line in Figure 8).

The mid‐line dose profiles received no dose between the gap of the legs, even though the fluence is not zero due to the padding for skin flash. The homogeneity of dose profiles with skin flash for both superior and inferior fields also reduced to within ±5% of the prescription dose except for the field junction area in the center where high‐frequency fluctuation existed. The surface dose computed in Eclipse for patients was between 96% to 98% prescription dose. It is worth noting that the surface dose calculated in TPS may not be accurate by the photon dose calculation algorithms. The skin dose is typically boosted by the placement of a beam spoiler close to the patient, which was not modeled yet in the current study. For the point dose at the center of the organ and the mean dose, the lung dose was always higher than the kidney dose, probably due to the low effective density and phantom scatter.

**FIGURE 10:**
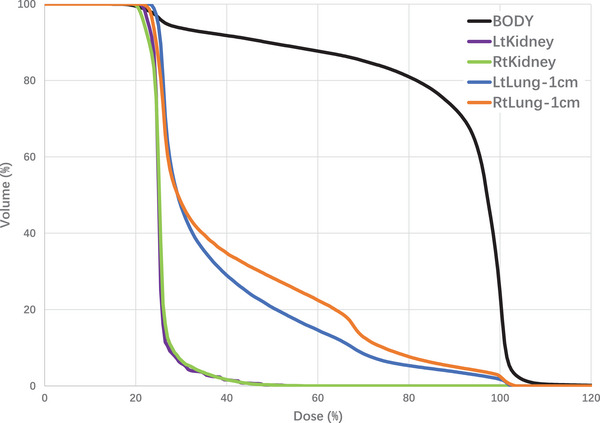
The dose volume histograms (DVH) for lungs‐1 cm, kidneys, and BODY for the IMRT plan.

Figure 10 shows the dose volume histogram (DVH) for lungs‐1 cm, kidneys, and body of the plan after the optimization. This mounts an additional benefit of the technique in that a full 3D dose distribution is available for plan evaluation and dose reporting, a substantial improvement over the previous techniques where only point‐based doses are available. Kidneys are well shielded due to the extra margin in the shielding design and fairly uniform tissue density nearby, therefore its doses are lower and uniform and close to the 25% of prescription dose limit. Lungs‐1 cm was derived from the actual lung contours to mimic the lung blocks, they have a larger deviation than the kidney because inner block margins do not align with Lungs‐1 cm exactly, and low‐density tissue and the phantom scatter. Nevertheless, a more accurate knowledge of the lung dose distribution is obtainable for further evaluation and potential revision in the block design. No extra processing was done on the BODY contours such as excluding the kidney, lungs, and the anterior and posterior tissues of lungs and kidneys receiving lower doses, this explains the lower dose regions (cold spot).

**FIGURE 11:**
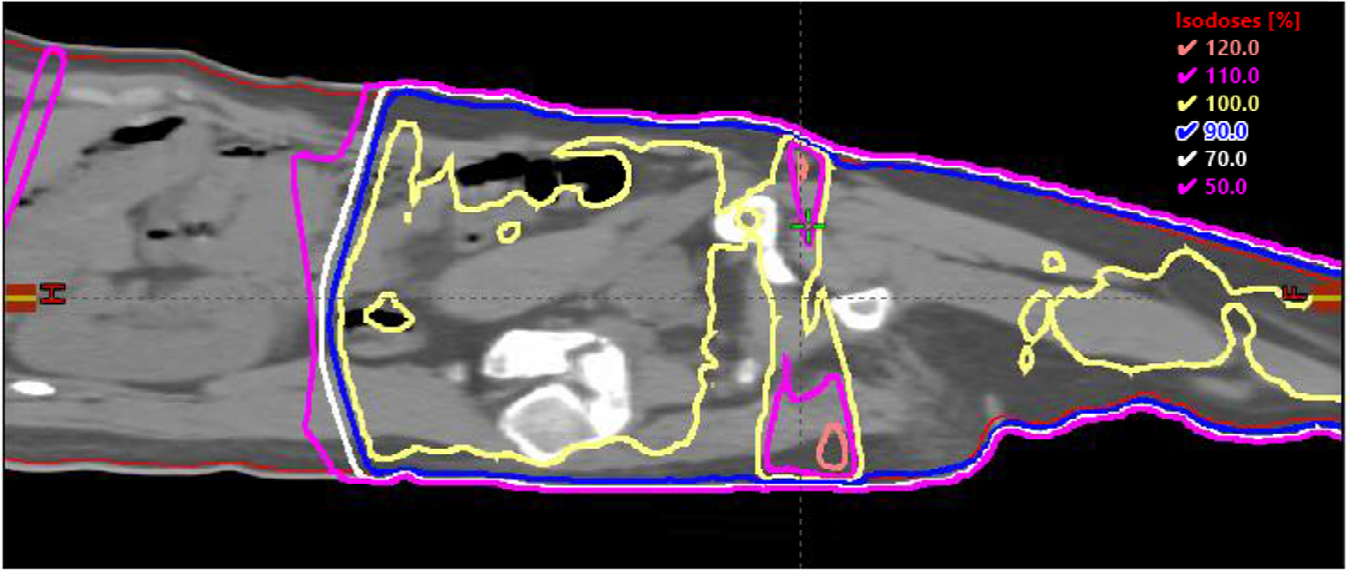
The isodose distribution on one sagittal plane near the field junction. The dashed black line is the boundary for superior and inferior fields. The left region with dose lower than 50% is the kidney region. Small hot spots of 120% dose are observed near the skin region.

Figure 11 shows the isodose distribution in one sagittal plane near the field junction region. Some hot spots of 120% near the skin region are observed. Kidney region on the left side is well spared.

**FIGURE 12:**
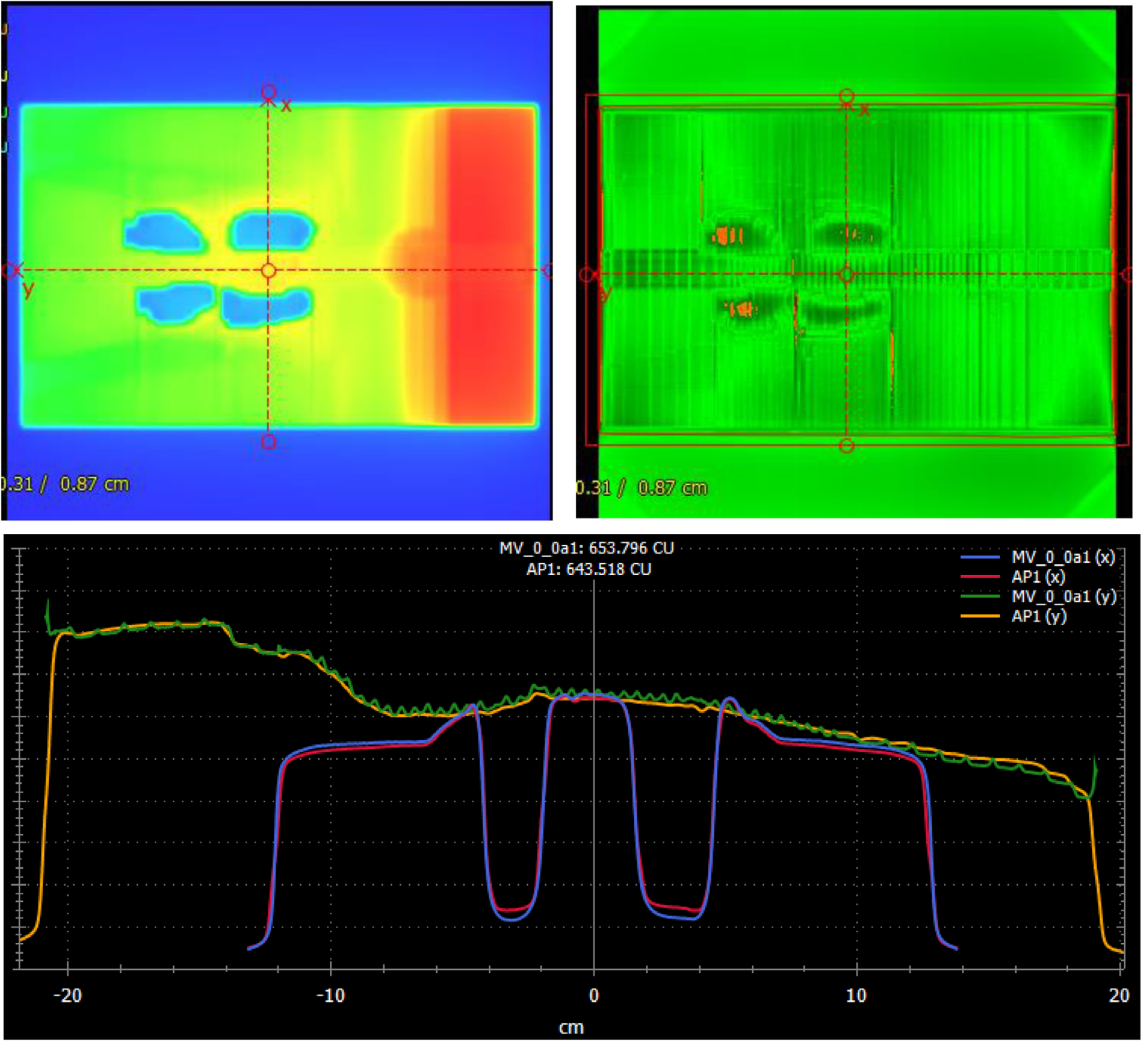
Portal dosimetry QA results for the beam AP1. Top left: predicted fluence map, the head is on right; Top right: the Gamm map between predicted and measured with criteria of 3% in dose difference, 2 mm in distance to agreement, and 10% in the threshold. Bottom: profile comparison for the two orthogonal red dashed lines in the top left plot. Red/Blue profiles represent the short vertical line that passes through the Lungs, and Yellow/Green lines represent the long horizontal line from head to umbilicus (the axis direction of Y is reversed due to a different coordinate system).

The portal dosimetry QA showed excellent results. The Gamma pass rate (percent of pixels with Gamma value <1.0) ranges from 98.1% to 98.6% for all four fields. Figure 12 shows the result for beam AP1. During portal dosimetry QA measurement, the EPID is placed normally to the beam entry, not at an oblique angle as in patient treatment, therefore, the fluence profile is not uniform as in the dose profile, which is clearly shown in the profile plot at the bottom. The ripples along the green line represent the MLC inter‐leaf leakage, about 2%. One point worth mentioning here is the portal dosimetry on a 40 × 40 cm^2^ MV EPID allows the full‐size fluence map to be verified, which is an advantage over other types of QA devices that may only be able to verify the partial fluence maps.

The clinical requirement for the dose rate is <15 cGy/min to the body. Given that the prescription dose is 100 cGy per direction (AP or PA) per fraction, the minimum treatment time is therefore 100/15 = 6.7 min, assuming that the machine dose rate can be continuously adjusted. In practice, the machine dose rate is only available in increments of 100 MU/min in clinical mode, for example, 100, 200, 600 MU/min, therefore the dose rate is typically rounded down to the next 100. In our typical IMRT plan, the AP1 (the superior field that covers both lungs and kidneys) has a MU of ∼2400, and AP2 (the inferior field that covers legs) has a MU of ∼1200. Both AP1 and AP2 have 2 split sub‐fields. This will translate into a dose rate of 2400/2/6.7, or ∼180 MU/min, and 1200/2/6.7, or ∼90 MU/min, to achieve the minimum treatment time of 6.7 min for each subfield, for a total of ∼27 min beam‐on time per direction, and ∼54 min for the whole treatment fraction. If the clinical protocol does not have a minimum dose rate requirement, that is, the linac can be operated at the highest dose rate available, such as 600 MU/min, then the treatment time can be much reduced, to (2400+1200)/600 = 6.0 min per patient position, and a possible 30 min overall treatment time including changing positions from supine to prone.

## 4 DISCUSSION

In this study, we developed an IMRT technique for TBI treatment at extended SSD. This technique is a natural extension of the standard AP/PA technique, with several advantages. First, this technique can be implemented in a standard linac room, without requiring it to be large enough to fit the patient in a single field, therefore, it is widely adaptable to many clinics. Second, the patient is in a comfortable recumbent position instead of the standing position, this is especially beneficial to many patients who are too frail to go through the long treatment time and therefore can improve patient safety during the treatment process. This also minimizes patient movement during treatment, thereby ensuring the quality of treatment delivery. The use of IMRT can effectively compensate the patient anatomy variations and improve significantly the dose uniformity inside the patient over the standard 3D technique with customized partial transmission blocks and compensators. Third, the elimination of these patient‐specific beam modifiers improves the efficiency of treatment planning and delivery, saving the labor‐intensive and time‐consuming process of fabricating and mounting these devices. Last, but not least, the use of a treatment planning system allows 3D dose distributions to be computed and potentially verified, thereby providing much more information about the treatment, for example, the dose volume histograms for lungs and kidneys allow much‐improved evaluation of the TBI treatment and can better correlate with the clinical outcome.

Before this technique of floor‐based recumbent IMRT technique was developed, quite a few alternatives were investigated. For example, placing the patient on the couch at extended SSD versus on the floor; fixed gantry with patient repositioning versus fixed patient position with multiple gantry angles. While the combination of them produces many options, and each has advantages and disadvantages, the technique that was presented stood out due to its practical value and general applicability.

A non‐standard IMRT optimization algorithm was developed for this technique, where there is no objective function. Rather, the only requirement is the uniform dose for the target, which is the whole body to be at the prescription dose, except the critical organs such as lungs and kidneys, whose prescription doses are also uniform, albeit at a much lower level (25% of the target prescription dose in the protocol^8^ used). Therefore, the goal is quite simple, with multiple uniform dose levels. In addition, the beam orientations are also fixed at AP and PA. Given all these requirements, the simple iterative fluence‐dose‐scaling method is adequate. As shown, only 2–3 iterations can reduce the dose uniformity to within 5% at the effective midplane. We want to point out that the in‐house software is used in this study to generate the optimal fluence. While the rest of the workflow is the same as all other treatment planning, such as leaf sequencing, dose calculation, plan evaluation, and pre‐treatment QA, extensive pre‐clinical studies and IRB approval will be needed before patient treatment can commence.

An important concept of effective mid‐plane was proposed, to account for the patient anatomy variations. This allows IMRT fields from AP and PA directions to be symmetric, with equal contributions to the dose, which is very similar to the standard 3D AP/PA technique.

In the current technique, the patient is CT scanned once in the Supine position but will be used in treatment planning for both Supine (for AP fields) and prone treatment positions (for PA fields). For the prone position, the planning and treatment gantry angles are not the same but symmetric along the patient midplane, as shown in Table 1 and Figure 1c. This way, the composite dose from all fields can be obtained easily for plan evaluation. Alternatively, one can flip the CT image during the planning of the prone position for PA fields, so the gantry angles will be the same for planning and treatment. The drawback is that the composite dose distribution will not be easily displayed in the planning system. Both methods are done under the assumption that the patient is rigid and maintains the same pose in Supine and Prone positions. Such assumptions are valid for large fields at extended SSDs. However, it is possible to have a separate CT for each position and they can be used for the planning. The current technique can accommodate the use of 2 CTs, because the optimization objective is for the dose on the patient effective midplane, and AP and PA fields are optimized independently.

In the phantom study, the lung was contoured and used in the plan optimization as critical organs, but it was not assigned a lower density to simulate the actual lung tissue, as the purpose was only to demonstrate that the optimization technique was able to reduce the fluence significantly to the critical organs and was able to account for the oblique gantry angles. This also facilitated dose measurement in the homogeneous phantom, that is, the lung structure was treated the same way in planning and measurement. In the patient planning study with actual CT images, there was no measurement performed, except pre‐treatment QA with portal dosimetry. The Anisotropic Analytical Algorithm (AAA) algorithm was used for the dose calculation, it is considered acceptable for RTOG clinical trials in the lung region since it can handle the lung material accurately, and it is also standard in our clinical practice. There is no density override in the lung region in the patient plan.

To ensure that the patient skin dose is adequate, a beam spoiler is used in many TBI techniques, which acts as a secondary scatter source closer to the patient. Although the beam spoiler was not included in the presentation of this study, it can and will be included in the clinical implementation of this technique.

As shown in Figure 11, there may be some hotspots near the field junction region. They are off the effective midplane and, therefore are not accounted for in the plan optimization process. Patient setup errors are likely to increase the hotspot, both in the dose level and the volume. If these hotspots are considered clinically significant, then actions must be taken to reduce them. One technique is the use of the brush in the fluence editing toolset to lower the fluence values near the boundary and re‐calculate the dose for evaluation, a few iterations usually are adequate to bring the hotspot under control, similar to practices in dose painting in breast IMRT planning. Keep in mind that the beam geometry in this technique is of the nature of a parallel‐opposed beam, and the relative dose profile is nearly constant along the beam direction, so the removal of the off‐midplane hotspot may create cold spot near the midplane, and trade‐offs must be considered.

To mitigate the effect caused by the setup error and to improve the robustness of the plan, two techniques can be explored. One option is the isocenter/junction shift. In this technique, the junction is shifted among a few positions a few cm apart, to spread out the hotspot regions and lower the cumulative hotspot value. So a few similar treatment plans with different junction points will be created, and one plan is used for one fraction with a single junction. For multiple‐fraction TBI treatment, this requires a bit extra effort at treatment planning, but with the same treatment delivery efficiency. However, this is not realistic for single‐fraction TBI treatment, because these different junction points require multiple patient setups and verifications. Other options such as feathering can be explored. In this technique, overlap between AP1 and AP2 (similarly PA1 and PA2) is needed. This will require the gantry angles to be changed. A 1˚ change (339˚→340˚ for AP1 & 21˚→20˚ for AP2) will lead to about 3.5 cm overlap at the patient midplane. This corresponds to about 2 pairs of MLC leaves at the edge of the field. Likewise, 2˚ will lead to an overlap of 7 cm. As a result, the maximum patient height/length is reduced by the same amount. During plan optimization, the algorithm will produce equal fluence in the overlap region. After the optimization, they will be post‐processed to produce the gradient profile (with the same sum as before). The idea is that feathering will make the treatment plan more robust to the setup errors and can reduce the hotspot inside the patient. The full investigation of these techniques is beyond the scope of this paper and will be the topic of future study.

A practical issue with this technique is the dose rate requirement. For example, as shown in the results section, to keep the target absorbed dose rate to be below 15 cGy/min, the machine dose rate can be a continuous value, such as 180 MU/min. This is not clinically available. To meet the requirement, it needs to round down to the next possible value of 100 MU/min and will prolong the treatment by almost a factor of 2, which may not be desirable. It is worth pointing out that this is not the limitation of the technique, but rather the linac software. For example, in Truebeam linac, the IMRT can be delivered in any arbitrary dose rate, but, only in research mode currently.

It is worth mentioning that many alternatives to the traditional technique of parallel‐opposed beams have been developed recently in the treatment of TBI, taking advantage of advancements in the Linac technology and treatment planning system, including the use of IMRT and VMAT. Some are dramatic departures from the tradition, in that patient is placed on the standard couch, and either VMAT (or helical Tomotherapy) or IMRT is used.^15^, ^23^, ^27^, ^28^, ^33^, ^34^ The advantages are that the sophisticated inverse planning system can be used to produce superior dose distributions, good immobilization, and comfortable patient position, the drawback includes long planning time, field junction involving multiple isocenters, and possible long treatment time. Some specially made devices may facilitate patient positioning.^21^, ^35^ Another type of development is the natural extension of the traditional 3D technique, with the patient placed at extended SSD treated by either VMAT or IMRT.^15^, ^16^, ^24^, ^36^, ^37^ The technique presented in this paper belongs to this category. They closely mimic the traditional technique, but produce much better dose distribution, eliminate labor‐intensive block fabrication process, and improve efficiency in planning and treatment delivery. The drawbacks include multiple patient positions during treatment which can affect the patient dosimetry.

## 5 CONCLUSIONS

In this study, we developed a promising IMRT technique for TBI at extended SSD. The technique evolves naturally from the current standard 3D AP/PA technique practiced at our clinic. The patient is in a comfortable and reproducible recumbent position and the technique can be executed in any size treatment room. The use of IMRT allows volumetric doses to be evaluated and recorded to correlate with clinical outcomes. It could achieve excellent dose homogeneity and sparing of lungs and kidneys without the labor‐intensive process of making blocks and compensators. We believe this technique has a lot of potential in clinical treatment.

## CONFLICT OF INTEREST STATEMENT

The authors declare no conflicts of interest.
